# Grazing-incidence X-ray diffraction of single GaAs nanowires at locations defined by focused ion beams

**DOI:** 10.1107/S0021889813004226

**Published:** 2013-06-07

**Authors:** Genziana Bussone, Rüdiger Schott, Andreas Biermanns, Anton Davydok, Dirk Reuter, Gerardina Carbone, Tobias U. Schülli, Andreas D. Wieck, Ullrich Pietsch

**Affiliations:** a Festkörperphysik, Universität Siegen, Walter-Flex-Strasse 3, D-57072 Siegen, Germany; b European Synchrotron Radiation Facility, F-38043 Grenoble, France; c Lehrstuhl für Angewandte Festkörperphysik, Ruhr-Universität Bochum, D-44780 Bochum, Germany

**Keywords:** semiconductor nanowires, growth, grazing-incidence X-ray diffraction, GaAs

## Abstract

Grazing-incidence X-ray diffraction measurements on single GaAs nanowires (NWs) grown on a (111)-oriented GaAs substrate by molecular beam epitaxy are reported. The positions of the NWs are intentionally determined by a direct implantation of Au with focused ion beams. This controlled arrangement in combination with a nanofocused X-ray beam allows the in-plane lattice parameter of single NWs to be probed, which is not possible for randomly grown NWs. Reciprocal space maps were collected at different heights along the NW to investigate the crystal structure. Simultaneously, substrate areas with different distances from the Au-implantation spots below the NWs were probed. Around the NWs, the data revealed a 0.4% decrease in the lattice spacing in the substrate compared with the expected unstrained value. This suggests the presence of a compressed region due to Au implantation.

## Abstract 2

The crystalline structure of single free-standing GaAs nanowires, grown by molecular beam epitaxy on a GaAs substrate at specific positions defined by focused ion beams, and the substrate regions close to the Au-implanted regions are investigated through grazing-incidence X-ray diffraction.

## 1. Introduction

Semiconductor nanowires are crucial for the conception of advanced electronic and photonic nanoscale devices (Yang *et al.*, 2010 ▶). Possible applications belong to the fields of photovoltaics (Law *et al.*, 2005 ▶), *e.g.* nanowire (NW) solar cells, and photonics and optoelectronics, *e.g.* semiconductor light-emitting diodes (Ponce & Bour, 1997 ▶). The crystal structure of nanowires suitable for many of those applications has to be homogeneous and free of defects. In addition, the control of their size and their exact location on the substrate is necessary. Those qualities strongly depend on the growth process. The typical fabrication methods for III–V semiconductor nanowires are the catalyst-assisted (Wagner & Ellis, 1964 ▶) and the self-assisted methods (Colombo *et al.*, 2008 ▶). In most cases, the NWs are grown with catalysts (frequently metals are used, *e.g.* Au) through the vapor–liquid–solid mechanism. In self-assisted growth, one constituent of the NW material replaces the metal in the seed function. For many practical devices, an arrangement of the NWs at intentional sites, for example in regular arrays, is required. In order to control the position of the wires accurately, techniques based on the pre-growth patterning of a catalyst mask material by electron beam lithography (Mårtensson *et al.*, 2003 ▶) and nanoimprinting lithography (Mårtensson *et al.*, 2004 ▶) have been successfully applied. These techniques require the use of a photoresist on the epi-ready monocrystalline surface, which represents a potential contamination source and cannot always be removed by cleaning. Therefore, a complete ultra-high-vacuum process, which includes layer growth, lateral structuration and subsequent NW growth, is preferable. That is why, here, the positions of the single NWs were defined by the direct implantation of Au using a focused ion beam (FIB) system (Gierak *et al.*, 2010 ▶). Depending on the growth parameters, two forms of crystal structure can be present in the wire: the cubic zinc blende and the hexagonal wurtzite phases. Phase-pure NWs can be obtained under specific growth conditions (Krogstrup *et al.*, 2010 ▶), but usually stacking faults and twins are present in the crystal structure (Dick *et al.*, 2010 ▶), and consequently influence the physical properties of the NW (Spirkoska *et al.*, 2009 ▶; De & Pryor, 2010 ▶; Jahn *et al.*, 2012 ▶). Therefore, a controlled growth on the nanoscale is crucial, as it allows the combination of different materials and the manipulation of the NW properties, opening the way to the design of new devices. As technological application demands the use of the nano-objects in their as-grown geometry on the substrate, one way to characterize the NWs without removing them from the substrate is high-resolution X-ray diffraction, which allows analysis of the defects in the crystal structure, phase compositions and strain. Previous characterization on single NWs using symmetrical and asymmetrical diffraction allowed determination of their morphology, structural composition and residual strain by combining the use of a focused X-ray beam with coherent diffraction imaging (Diaz *et al.*, 2009 ▶; Biermanns *et al.*, 2012 ▶). Additional details about the NW crystal structure can be obtained by probing the planes whose normal is perpendicular to the growth direction, along the height of the wire. This requires working under grazing-incidence X-ray diffraction (GID) conditions (Dosch *et al.*, 1986 ▶; Davydok *et al.*, 2012 ▶). In order to perform GID characterization on single NWs, the controlled arrangement of the NWs is critical; indeed, for randomly grown NWs the wide footprint of the X-ray beam on the sample does not allow the selection and analysis of a single wire, and therefore only average properties can be accessed. In this paper, the position-controlled growth of free-standing (111)-oriented GaAs nanowires and their characterization through grazing-incidence in-plane X-ray diffraction are presented.

## 2. Sample preparation

**Figure 1:**
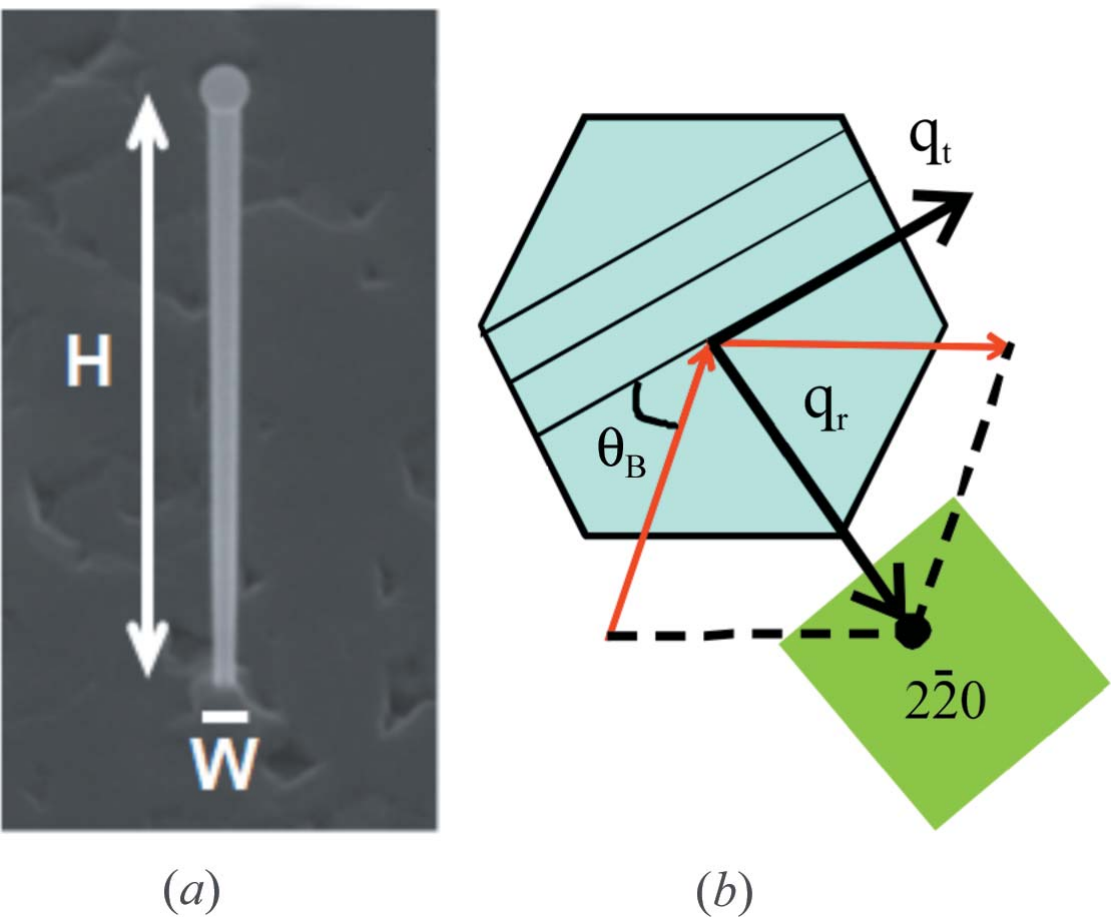
(*a*) A GaAs NW with growth axis parallel to the surface normal: diameter (W) ≃ 150 nm and height (H) ≃ 6 µm. (*b*) Top view of the GID geometry for a single NW for reflection 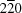.

**Figure 2:**
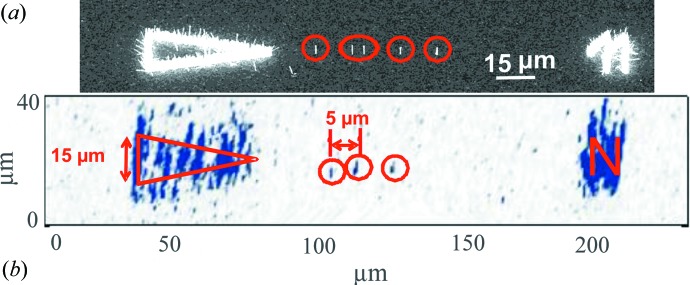
Comparison between the SEM image along a row of NWs (*a*) and a real space map (*b*) along a similar row under the diffraction condition for the GaAs symmetric 111 reflection. The diffraction signals of a triangular and a number-shaped marker (hard to identify in the RSM) are clearly visible. In the space between the markers, a few nanowires having the expected spacing of 5 µm can be distinguished. The positions of the NWs are marked with red circles.

**Figure 3:**
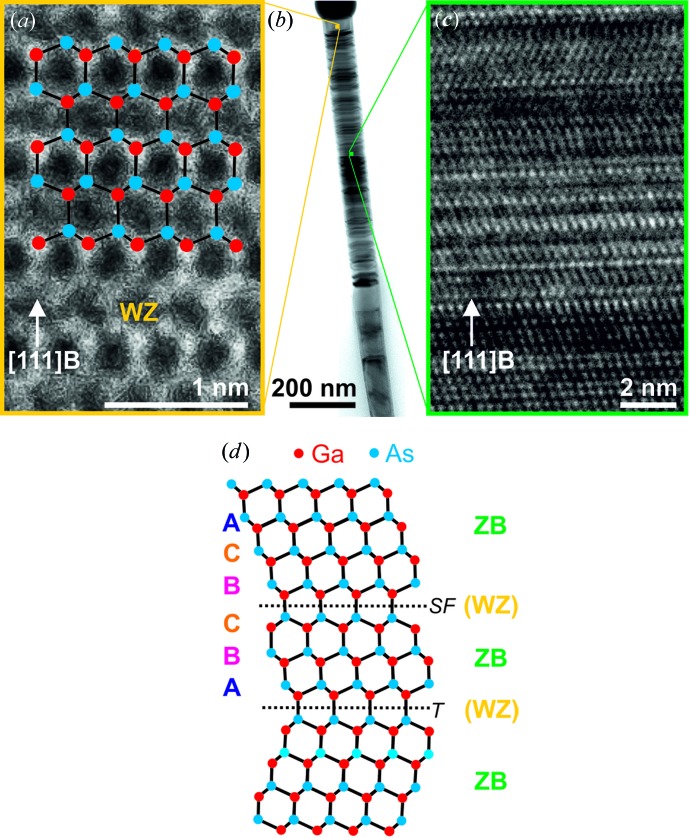
TEM images of a typical GaAs nanowire: (*a*) HRTEM image of a pure wurtzite (WZ) structure region with a simulation of the atom positions overlaid; (*b*) bright-field image of the top part of the NW; (*c*) HRTEM image of a zinc blende structure (ZB) region with twin planes (T) and stacking faults (SF); (*d*) schematic diagram of the atomic stacking for zincblende structure with twin planes and stacking faults as shown in (*c*).

The growth method consists in the well known Au-assisted vapor–liquid–solid molecular beam epitaxy (MBE) growth of GaAs nanowires combined with the capability of a FIB system to implant Au ions masklessly as ‘catalytic’ metal seeds at the desired sites. The GaAs nanowires were grown on GaAs(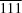) [or (111)B] substrates using a Riber EPINEAT V/III S MBE system, equipped with a Ga effusion cell and an As cracker source. First, the substrate surface was deoxidized at 913 K, and then a GaAs buffer layer was grown. Afterwards, the sample was transferred in vacuum to a FIB system, an Orsay Physics Canion 31-Z FIB column including an E × B mass filter. Single-charged Au ions were generated from an AuBeSi liquid alloy ion source. Defined dot patterns were implanted using an acceleration voltage of 30 kV. The typical penetration depth of the Au^+^ ions was estimated to be ∼15 nm with *SRIM* (Biersack & Haggmark, 1980 ▶; Ziegler *et al.*, 1985 ▶). It turned out that an ion fluence of around 10^6^ ions per spot was needed to obtain the growth of only one NW per site, although occasionally no or multiple NWs were obtained at one spot. The approximate size of the implantation spot in this case is ∼200 nm. The sample was transferred back to the MBE system and heated to 823 K. Then the NW growth was initiated without delay and performed for 60 min with an As_4_ flux to provide a constant V/III flux ratio of 13 and a Ga flux to produce a two-dimensional growth rate of 0.2 nm s^−1^ on GaAs(100). This resulted in (111)-oriented GaAs NWs with diameters of ∼150 nm and a height of ∼6 µm (Fig. 1 ▶ *a*), which were located along a straight line, with a separation of 5 µm from each other (Fig. 2 ▶ *a*). The orientation of the line of NWs with respect to the crystallographic directions of the substrate was chosen in such a way that the cubic 220 reflections of the NW and substrate could be accessed without intersection of the X-ray beam with more than one NW. The MBE growth parameters that were used for NWs and GaAs layers vary in temperature and As_4_ flux. GaAs layers grown with NW growth conditions (substrate temperature: 823 K; V/III flux ratio: 13) get a rough surface morphology. Because the layer growth does not stop while the NWs are growing, the (111)B surface between the NWs is typically fairly rough. This can be clearly seen in scanning electron microscopy (SEM) images (Fig. 1 ▶ *a*). The NW structure was determined with transmission electron microscopy (TEM). A mix of zinc blende and wurtzite structures was observed. The top part of a typical NW from the investigated NWs is shown in Fig. 3 ▶(*a*). In a high-resolution TEM (HRTEM) image (Fig. 3 ▶ *a*) the wurtzite structure is clearly visible. Fig. 3 ▶(*c*) shows an HRTEM close-up with zinc blende structure, twin planes and stacking faults, as is illustrated in a schematic diagram of the atomic stacking in Fig. 3 ▶(*d*).

## 3. Experimental method

**Figure 4:**
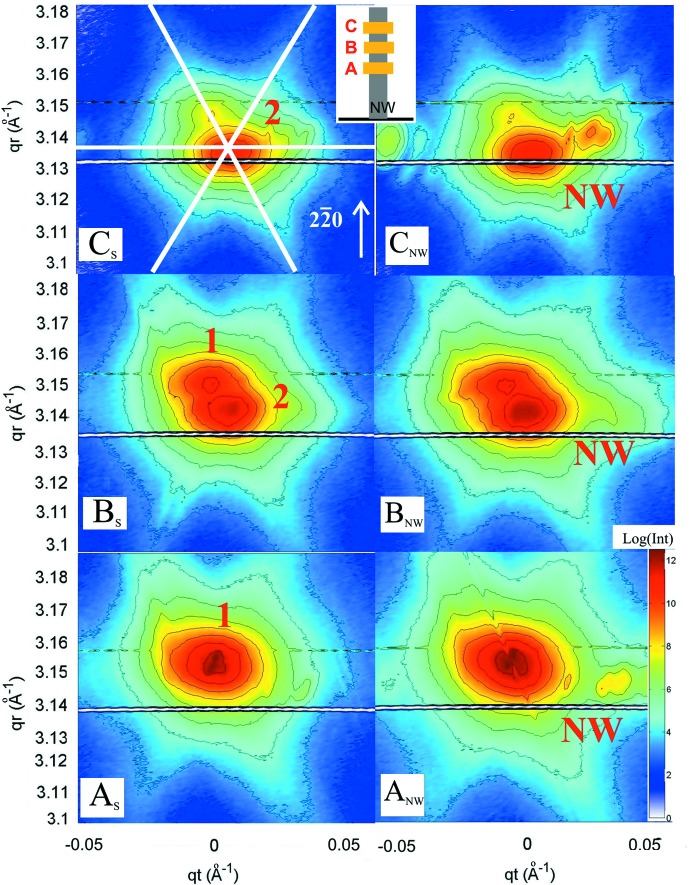
On the left, reciprocal space maps collected for three different regions of the substrate (A_S_, B_S_ and C_S_) at different distances from the NW. The regions have dimensions 86 µm × 500 nm, and are located ∼570 µm from each other along the direction perpendicular to the NW row. The strained and unstrained diffraction peaks are named 1 and 2, respectively. On the right, reciprocal space maps collected for regions A_NW_, B_NW_ and C_NW_ along the NW. The NW signal, indicated in red as ‘NW’, is clearly visible on the right of the substrate peak.

The NWs were investigated in grazing-incidence geometry with an 8 keV nanofocused X-ray beam at beamline ID01 at the ESRF in Grenoble, France. The required spatial resolution was obtained with a circular Fresnel zone plate (Jefimovs *et al.*, 2007 ▶), giving a measured focal size of 300 × 500 nm (vertical × horizontal) (Diaz *et al.*, 2009 ▶). Owing to the GID geometry, for an incident angle of 0.25°, the footprint of the X-ray beam on the sample surface was 68 µm along the direction of the X-ray beam propagation. A circular beam stop and a circular order-sorting aperture, positioned as close as possible to the sample, were used to block the transmitted beam and to clean the higher diffraction orders. First, the sample was aligned at the symmetric 111 Bragg reflection in order to map out the intensity distribution in the **x** and **y** directions of the sample. Here we considered that the NW may grow with a high content of the hexagonal wurtzite phase, which has a slightly larger out-of-plane lattice parameter compared with the substrate zinc blende reflection (Biermanns *et al.*, 2009 ▶). Therefore, we fixed the scattering angles at the position expected for wurtzite 0002 and scanned the sample along the **x** and **y** directions through the probing X-ray beam. Positions of increased diffracted intensity were identified as single NWs (Diaz *et al.*, 2009 ▶; Mocuta *et al.*, 2008 ▶). In Fig. 2 ▶(*b*) we show a sample area containing two markers and a row of single NWs spaced about 5 µm apart. The markers are already visible in an optical microscope, whereas the NWs could be identified only by the increased X-ray intensity. Moreover, only a few of the arranged NWs are visible along the line, but other places are empty, either because of missing NW positions in the implanted pattern or because of a small misalignment of the NWs with respect to the substrate. Subsequently, the identified NWs were analyzed using GID, focusing the X-ray beam onto the identified *x*, *y* positions along the specimen. Reciprocal space maps (RSMs) were collected around the zinc blende 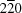 reflection (equivalent to the wurtzite 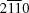 reflection) at different heights along the NW. Because the NW diameter is smaller than the X-ray beam and the absorption by the NW is negligible, most of the beam still hits the substrate at different distances from the NW position. The components of the scattering vector **q** are defined as *q*_r_ and *q*_t_, respectively parallel and perpendicular to the normal of the (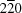) planes shown in Fig. 1 ▶(*b*). With the two-dimensional MAXIPIX detector (pixel size of 55 × 55 µm; Ponchut *et al.*, 2007 ▶), we mapped the complete two-dimensional region in reciprocal space around the diffraction peak. Reciprocal space maps were calculated by integrating the diffracted intensity along the whole range of the exit angle. For NWs grown epitaxially on a substrate, the lattice planes of both substrate and NW can be probed in GID scattering geometry. If the NW and substrate are made of different materials, they diffract at different Bragg angles θ_B_ and their signals can easily be separated. In the present case, the NW and substrate are both made of GaAs, *i.e.* they diffract at the same Bragg angle. However, by carefully examining the detector images, an additional signal, observed only for scans performed at NW positions and therefore attributed to the NW contribution, can be seen at a slightly different angular position along the rocking scan compared with the GaAs substrate. This allows distinction between signals originating from the GaAs substrate and the NW signal with much lower intensity, because of their different *q*_t_ positions, Δ*q*_t_ = 0.035 (2) Å^−1^. This separation is probably caused by a twist of the NW growth axis with respect to the substrate normal (Keplinger *et al.*, 2009 ▶). This may also explain the fact that other NWs could not be found at the expected position if their misorientation exceeds a certain value. Fig. 4 ▶ shows RSMs measured for selected regions on the samples along the NW line shown in Fig. 2 ▶(*a*): the graphs labeled A_S_, B_S_ and C_S_ refer to a region free from NW growth; the graphs labeled A_NW_, B_NW_ and C_NW_ are relative to one of the identified NW positions. The intensity of the substrate is much higher than that of the NWs owing to the large difference in scattering volumes.

## 4. Results

**Figure 5:**
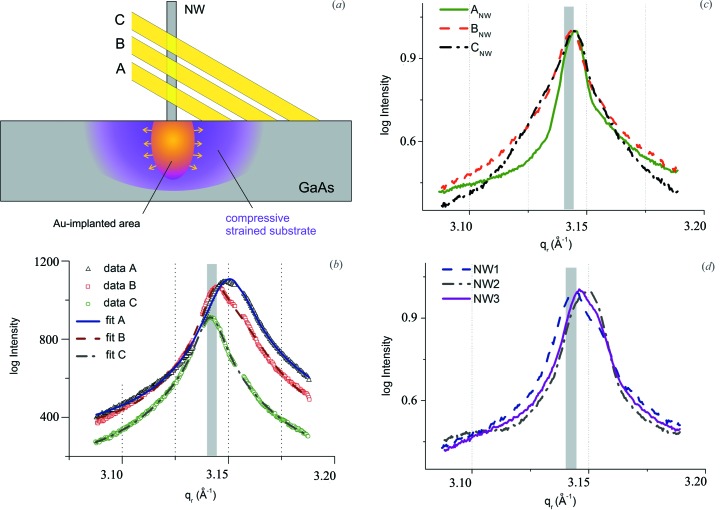
(*a*) A sketch of the NW and the substrate probed around the Au-implanted area. (*b*) Integrated diffracted intensity as a function of *q*_r_ for the three analyzed regions in the substrate. Experimental and fitted data are shown. Here, and in the following graphs (*c*) and (*d*), the value of the uncompressed GaAs (3.142 ± 0.002 Å^−1^) is represented by a gray rectangular area. (*c*) Normalized integrated diffracted intensity as a function of *q*_r_ for three different positions (A, B and C) along the same NWs. (*d*) Normalized integrated diffracted intensity as a function of *q*_r_ for three different NWs, at position B.

By changing the vertical position of the NW with respect to the incident beam, *i.e.* measuring at different heights along the NW, changes of the NW signal were hard to identify. However, major changes were observed in the substrate signal, because the position of the incident beam on the sample is modified simultaneously (Fig. 5 ▶ *a*). Fig. 4 ▶ shows reciprocal space maps taken at different lateral positions on the substrate, marked as A_S_, B_S_ and C_S_, and corresponding to the three different positions of illumination along the NW long axis, A_NW_, B_NW_ and C_NW_. Considering steps of 2.5 µm in the vertical direction, the illuminated areas are separated laterally by 570 µm each, where position A is the closest and position C the most distant from the NW (Fig. 5 ▶ *a*). The substrate signals show two different features partially overlapping for region B, at *q*_r_ = 3.156 (2) Å^−1^ [indicated as 1 in Fig. 4 ▶ (A_S_, B_S_)] and at 3.142 (2) Å^−1^ [indicated as 2 in Fig. 4 ▶ (B_S_, C_S_)], their intensity ratio changing as a function of illumination position. At all positions, a ‘hexagonal star’ [marked with white lines in Fig. 4 ▶ (C_S_)] was observed, probably originating from crystalline grains within the GaAs layer growing simultaneously with the NW. A few additional artefacts were visible close to the Bragg peaks, probably due to randomly oriented GaAs crystallites distributed on the substrate surface. They were also observed in SEM images and might have been created during the cleavage of the as-grown sample into smaller pieces. For quantitative evaluation, line profiles (Fig. 5 ▶ *b*) were extracted from the RSMs shown in Fig. 4 ▶ (A_S_, B_S_ and C_S_) along the radial direction *q*_r_. As mentioned above, two different peaks can be identified. For position A, peak 1 dominates. The two peaks show rather similar intensity at position B and peak 1 almost disappears at position C. The appearance of two different peak positions in *q*_r_ indicates the presence of a strained region. Considering peak 2 as the position of the unstrained GaAs, peak 1 indicates the presence of compressively strained GaAs in the vicinity of the implanted region. Indeed, the unstrained peak shows increasing intensity with increasing distance above the substrate (see Fig. 4 ▶ peaks relative to positions B_S_ and C_S_). At first glance, this interpretation might contradict the expectation. As a result of Au implantation, a lattice expansion is expected because the atomic radius of Au is always larger than that of Ga and As. However, the induced lattice damage might be so large that this region does not contribute to the Bragg scattering signal. On the other hand, an increased lattice parameter in the implanted core must result in an outer laterally compressed region of GaAs, which explains our finding. This compressed part dominates while probing the region close to the NWs, and we expect the compressive strain to decay slowly over a large distance from the implanted area. Because of the large footprint of the X-ray beam on the sample surface, the strained peak measures a mean strain value, which was determined by fitting the measured diffraction profile with two Lorentzians, one for the uncompressed and one for the compressed substrate contributions, shown by solid lines in Fig. 5 ▶(*b*). The data show a decrease of the in-plane lattice parameter: the region closer to the NW is compressed by 0.4% compared to the unstrained one. The NW signal is hardly accessible since the substrate is made of the same material; however there is evidence for the existence of NWs which can be further processed. A comparison between the different regions along one NW was carried out. As shown in Fig. 4 ▶ (A_NW_, B_NW_ and C_NW_), different regions along the NW were investigated by translating the sample along the growth axis with steps of 2.5 µm. In Fig. 4 ▶ (A_NW_), the compressed substrate peak and the NW signal can be observed at similar *q*_r_ values, but with Δ*q*_t_ = 0.035 (2) Å^−1^. By extracting the line profile along *q*_r_ of a selected region of interest around the NW signal (Fig. 5 ▶ *c*), it is observed that the signals coming from regions B_NW_ and C_NW_ along the NW are located at *q*_r_ positions similar to that found for the unstrained substrate. This means that the NW lattice is not strained. Conversely, the NW signal visible in A_NW_ appears shifted towards lower *q*_r_ by less than 0.03%; however an incomplete separation from the substrate cannot be excluded. As shown in Fig. 5 ▶(*d*), the profiles of RSMs along *q*_r_ were processed for three different NWs measured in this experiment for the middle position B. The curves are the same within the uncertainties of the experiment. This proves that we are able to separate the intensity pattern of an individual NW.

## 5. Conclusions

We have shown that individual free-standing GaAs NWs prepared by MBE after Au spot implantation by FIB into a GaAs substrate can be investigated by X-ray grazing-incidence diffraction using a nanofocused X-ray beam. We detected the presence of two diffraction peaks in different regions of the substrate. One corresponds to the unstrained substrate away from the NWs (more than 570 µm apart); the second peak originates both from the substrate deformation in the vicinity of the Au-implanted area and from the non­deformed region in the substrate. The implanted region itself could not be accessed. From the relative peak shift observed in these two distinct regions in the substrate, we conclude that the region close to the implanted area experiences an average lattice compression of 0.4% compared to the unstrained GaAs lattice. The detection of NWs grown within the implanted area was successful. For the first time, single NWs were investigated at different positions along their height. Within the uncertainty of the experiment, the data show that the lattice parameter of the NW equals that of the unstrained substrate. The wires show a certain twist of the growth axis with respect to the substrate normal, which made their identification challenging.
